# Cyanidin-3-O-glucoside and Peonidin-3-O-glucoside-Rich Fraction of Black Rice Germ and Bran Suppresses Inflammatory Responses from SARS-CoV-2 Spike Glycoprotein S1-Induction In Vitro in A549 Lung Cells and THP-1 Macrophages via Inhibition of the NLRP3 Inflammasome Pathway

**DOI:** 10.3390/nu14132738

**Published:** 2022-06-30

**Authors:** Warathit Semmarath, Sariya Mapoung, Sonthaya Umsumarng, Punnida Arjsri, Kamonwan Srisawad, Pilaiporn Thippraphan, Supachai Yodkeeree, Pornngarm Dejkriengkraikul

**Affiliations:** 1 Department of Biochemistry, Faculty of Medicine, Chiang Mai University, Chiang Mai 50200, Thailand; warathit_semmarath@cmu.ac.th (W.S.); srmapoung@gmail.com (S.M.); punnida_dream@hotmail.com (P.A.); k.srisawad@gmail.com (K.S.); tipprapant@gmail.com (P.T.); yodkeelee@hotmail.com (S.Y.); 2 Center for Research and Development of Natural Products for Health, Chiang Mai University, Chiang Mai 50200, Thailand; sonthaya.u@cmu.ac.th; 3 Department of Veterinary Biosciences and Veterinary Public Health, Division of Veterinary Preclinical Sciences, Faculty of Veterinary Medicine, Chiang Mai University, Chiang Mai 50100, Thailand; 4 Anticarcinogenesis and Apoptosis Research Cluster, Faculty of Medicine, Chiang Mai University, Chiang Mai 50200, Thailand

**Keywords:** black rice, anthocyanins, C3G, P3G, spike glycoprotein S1, COVID-19, post-acute COVID, anti-inflammation, NLRP3 inflammasome

## Abstract

Black rice is a functional food that is high in anthocyanin content, primarily C3G and P3G. It possesses nutraceutical properties that exhibit a range of beneficial effects on human health. Currently, the spike glycoprotein S1 subunit of SARS-CoV-2 (SP) has been reported for its contribution to pathological inflammatory responses in targeting lung tissue and innate immune cells during COVID-19 infection and in the long-COVID phenomenon. Our objectives focused on the health benefits of the C3G and P3G-rich fraction of black rice germ and bran (BR extract) on the inhibition of inflammatory responses induced by SP, as well as the inhibition of NF-kB activation and the NLRP3 inflammasome pathway in an in vitro model. In this study, BR extract was identified for its active anthocyanins, C3G and P3G, using the HPLC technique. A549-lung cells and differentiated THP-1 macrophages were treated with BR extract, C3G, or P3G prior to exposure to 100 ng/mL of SP. Their anti-inflammatory properties were then determined. BR extract at concentrations of 12.5–100 μg/mL exhibited anti-inflammation activity for both A549 and THP-1 cells through the significant suppression of *NLRP3*, *IL-1β*, and *IL-18* inflammatory gene expressions and IL-6, IL-1β, and IL-18 cytokine secretions in a dose-dependent manner (*p* < 0.05). It was determined that both cell lines, C3G and P3G (at 1.25–10 μg/mL), were compatibly responsible for the significant inhibition of SP-induced inflammatory responses for both gene and protein levels (*p* < 0.05). With regard to the anti-inflammation mechanism, BR extract, C3G, and P3G could attenuate SP-induced inflammation via counteraction with NF-kB activation and downregulation of the inflammasome-dependent inflammatory pathway proteins (NLRP3, ASC, and capase-1). Overall, the protective effects of anthocyanins obtained from black rice germ and bran can be employed in potentially preventive strategies that use pigmented rice against the long-term sequelae of COVID-19 infection.

## 1. Introduction

The risk of severe acute respiratory syndrome coronavirus 2 (SARS-CoV-2) infection is marked by higher levels of proinflammatory cytokines in the patient’s blood, including IL-1β, IL-6, IL-18, TNF-α, MCP-1, and IFN-γ, which have been reported in infected patients with severe symptoms [1,2]. These clinical and laboratory traits are linked to hyper-inflammation and are associated with an activation of the innate immune system [3]. While innate immune response is essential for anti-viral host defense, excessive inflammatory cytokines are cytotoxic for respiratory epithelial cells and vascular endothelial cells [4]. During SARS-CoV-2 infection, there is an exaggerated immune response that resulted in an uncontrolled release of pro-inflammatory cytokines. This status is also known as the “cytokine storm” effect [5,6]. The cytokine storm results in harmful damage to lung tissues. Additionally, the pathology of SARS-CoV-2 infected lungs is further worsened by the innate immune cells, such as macrophages, since the immune cells are activated by viral components and products of dead cells that could trigger the vicious cycle of inflammatory responses [7,8].

At present, the pathogenesis of inflammation-related SARS-CoV2 infection has not been fully elucidated but has been found to be potentially associated with the major structural proteins of SARS-CoV-2 (spike protein) [3,9]. Interestingly, during SARS-CoV-2 infection, the binding of the spike protein to the infected cells (mainly lung epithelial cells and immune cells) can activate different pathways rather than a simple entry to the host cell. Spike protein can also do additional damage by activating toll-like receptors (TLRs) leading to the secretion of pro-inflammatory cytokines independent of viral entry [10]. Furthermore, activated TLRs involve multiple inflammatory signaling pathways, such as MAPK, JAK-STAT, and NLRP3 inflammasome, as well as activate transcription factors, such as NF-κB, and AP1, that are known to regulate the expression of the genes involved in immunity and inflammation in different cells [7,11].

Presently, many in vitro, in vivo, and clinical studies have targeted the NLRP3 inflammasome-inflammatory pathway as a potential immunological intervention against SARS-CoV-2 infection, specifically as an attenuation of inflammatory responses [12,13]. The unregulated NLRP3 inflammasome pathway causes the release of pro-inflammatory molecules including IL-1β and IL-18 to further expand the host immune response, which is usually correlated with multiple disease conditions including gout, autoinflammatory diseases, and the inflamm-aging phenomenon in the aging population, and the prolonged sub-chronic inflammation condition, frequently observed 2–3 months after the SARS-CoV-2 infection, that is referred to as post-acute COVID syndrome or long-COVID [4,6,14,15].

Recently, pigmented rice varieties have received increased attention for the various phytochemical compounds including phenolics, flavonoids, proanthocyanidins, and anthocyanins. These bioactive compounds are primarily located in the outer layer parts such as in the germ, or bran [16,17,18]. Black rice germ and bran are characterized by high amounts of anthocyanins, a subclass of flavonoid phytochemicals that include cyanidin-3-o-glucoside (C3G) and peonidin-3-o-glucoside (P3G) [19,20]. These phytochemicals have been reported for their antioxidant capacity and anti-inflammatory activity [19,21]. Additionally, the nutritional benefits of pigmented rice varieties have been reported to be much more beneficial than those of white rice [22]. Previously, anthocyanin-rich black rice extract was found to exhibit anti-inflammatory effects on the macrophage cell line and could attenuate the inflammatory response under lipopolysaccharide (LPS)-induced conditions [23]. Additionally, the synergistic effect of the black rice germ and bran supplement, and exercise program intervention, demonstrated a sustainable improvement in physical performances, and the modulation of inflammatory and endocrine biomarkers (IL-6, CRP, and IGF-1) in the aging population [24].

With regard to SARS-CoV-2, anthocyanins have been previously investigated as the candidate for anti-SARS-CoV-2 agents using in silico studies. The molecular docking approach of cyanidin and peonidin, a subclass of the anthocyanins derived from berries, were demonstrated as effective anti-SARS-CoV-2 agents as they exhibited low binding energy with the main protease and targeted the receptor-binding domain (RBD) of spike protein for SARS-CoV-2 [25]. Moreover, 10 anthocyanins, including C3G, were screened using in silico approaches to determine the structural relationship activity of anthocyanins that could inhibit 3CLpro of SARS-CoV-2 and could be recognized for their potent effect in inhibiting viral infection by reducing the pathogenicity of the virus [26]. Nevertheless, there is a limit number of studies that examined the effects of spike protein on inflammatory responses in in vitro models. Moreover, the potential benefits of functional foods such as black rice germ and bran, and active anthocyanins (C3G and P3G) as the anti-inflammatory agents for SARS-CoV2-induced inflammation, have not been investigated yet.

Therefore, it would be very intriguing to investigate the inhibitory effects of the anthocyanin-rich fraction of black rice germ and bran and the active compounds in the hallmark cells involved with inflammation during SARS-CoV-2 infection. The objectives of this study were to investigate the anti-inflammatory properties of the C3G and P3G-rich fraction of black rice germ and bran by determining the inhibition of SP-induced inflammatory gene expressions and cytokine secretions and to determine their responsible anti-inflammatory pathway using the in vitro models (A549 lung epithelial cells and THP-1 macrophages). The results obtained from this study could potentiate changes in the consumption of pigmented rice as a nutritional source and plant-based medicine.

## 2. Materials and Methods

### 2.1. Chemical and Reagents

Dulbecco’s Modified Eagle Medium (DMEM) was purchased from Gibco (Grand Island, NY, USA). Roswell Park Memorial Institute (RPMI)–1640 medium (R8758, Sigma-Aldrich), Cyanidin-3-O-glucoside (C3G), peonidin-3-O-glucoside (P3G), phorbol 12-myristate 13-acetate (PMA), and anti b-actin were purchased from Sigma-Aldrich (St. Louis, MO, USA). Fetal bovine serum (FBS) was purchased from Thermo Scientific (Waltham, MA, USA). Recombinant human coronavirus SARS-CoV-2 Spike Glycoprotein S1 (Active) (ab273068) was purchased from Abcam (Cambridge, UK). TRI reagent^®^ was purchased from Merck Millipore (Billerica, MA, USA). ReverTra Ace^®^ qPCR Master Mix was purchased from Toyobo Co., Ltd. (Osaka, Japan). SensiFAST^TM^ SYBR^®^ Lo-ROX Kit was purchased from Meridian Bioscience^®^ (Cincinnati, OH, USA). The anti-NLRP3, anti-ASC, anti-caspase-1, primary antibody, and horseradish peroxidase-conjugated anti-mouse- or anti-rabbit-IgG were purchased from Cell Signaling Technology (Danvers, MA, USA).

### 2.2. Herb Materials

Black pigmented rice (*Oryza sativa* L.) or Kum Doi Saket glutinous rice was harvested in wet season from Doi Saket Organic Farm, Chiang Mai Province, Thailand. A voucher specimen number (023149) was certified by the herbarium at the Flora of Thailand, Faculty of Pharmacy, Chiang Mai University, Thailand.

### 2.3. Preparation of C3G and P3G-Rich Fraction of Black Rice Germ and Bran

The extraction method was followed from our previously described protocol [23]. Briefly, one kilogram of whole grain black rice was milled with the rice milling machine to obtain the germ and bran. Then, the germ and bran were collected and soaked in 50% ethanol for 24 h. The rice samples were then filtered through filter paper to separate the residue. The filtered samples were then evaporated using a rotary vacuum evaporator (BUCHI, Flawil, Switzerland) to obtain the ethanolic fraction. The fraction was further partitioned with saturated butanol using a separating funnel. The upper layer part in the butanol fraction was collected to obtain a medium polar fraction, while the residual fraction or lower part was discarded. The medium polar fraction was then evaporated to remove the butanol and it was freeze-dried to obtain the C3G and P3G-rich fraction of black rice germ and bran (BR extract). The fraction was kept at −20 °C for further experiments.

### 2.4. Total Phenolic Content and Total Flavonoid Content

The total phenolic content of herbal extracts used in this study was determined by the modified Folin−Ciocalteu assay as previously described [27]. The total phenolic content was calculated from the absorbance of the mixture measured at 765 nm using a UV-visible spectrophotometer and then compared with the standard gallic acid (GA) and was shown as milligrams of GA equivalents per gram of extract (mg GA/g extract).

The total flavonoid contents were measured using the aluminum chloride (AlCl_3_) colorimetric assay with slight modifications [28]. The absorbance of the mixture was measured at 510 nm using a spectrophotometer and then compared with the standard catechin. The total flavonoid content was expressed as milligrams of catechin equivalents per gram of extract (mg catechin/g extract).

### 2.5. Total Anthocyanin Content

Total anthocyanin content was measured using a pH differential method [29]. The BR extract was dissolved in 0.1% HCl in 80% methanol and incubated for 12 h. The appropriate dilution factor was first determined before the experiment by diluting the test portion with pH 1.0 buffer until absorbance at 520 nm was within the linear range of the spectrophotometer (between 0.2 and 1.4 AU.). Then, the two dilutions of BR extract solution were prepared for each developing stage (two dilutions of the test sample, each dilution containing 1 part test sample portion, and 4 parts buffer so as not to exceed the buffer capacity of the reagents). The first sample (0.25 mL) was diluted with 1 mL of 0.025 M potassium chloride (KCl) buffer pH = 1.0 and the second was diluted with 1 mL of 0.45 M sodium acetate (CH_3_COONa) buffer pH = 4.5. The samples were incubated at room temperature for 15 min. Then, the absorbance of the sample was measured at 520 and 700 nm using UV−visible spectrophotometer. The total anthocyanin content was calculated using the following formula:

(1) $$Total\;anthocyanin\;content=\frac{A\times Mw\times DF\;\times 10^{3}}{\varepsilon \times 1}$$

*A* = (*A*_520nm_ − *A*_700nm_) pH 1.0 − (*A*_520nm_ − *A*_700nm_) pH 4.5
*MW* = molecular weight of C3G
*DF* = dilution Factor
*ε* = molar extinction coefficient = L × mol^–1^ × cm^–1^
L= cell path length (1 cm)

### 2.6. Identification of Active Anthocyanin Compounds in BR Extract Using High-Performance Liquid Chromatography (HPLC) Technique

Analysis of anthocyanin compounds (C3G and P3G) in the BR extract was performed using HPLC technique (Infinity 1260, Agilent Technologies, Santa Clara, CA, USA) using reversed-phase C18 column (Zorbax Eclipse Plus C18, 250 mm × 4.6 mm, 5 µm, supplied from Agilent Technologies, Santa Clara, CA, USA). The HPLC protocol was followed from a previously described method [30]. The mobile phase was composed of mobile phase A (0.4% trifluoroacetic acid in distilled water) and mobile phase B (0.45% trifluoroacetic acid in acetonitrile) under isocratic conditions. The detection wavelength was at 520 nm. The flow rate was set to 1.0 mL/min for 30 min. The temperature of the column was set at 40 °C. The injection volume was 10 μL. The concentrations used for HPLC analysis were as follows: BR extract at 10,000 μg/mL and 20,000 μg/mL; C3G and P3G standards at the range of 0–100 μg/mL. The peak area was calculated and compared with the standard to establish the concentration for each detected compound (mg/g extract).

### 2.7. Cell Cultures

A549 lung epithelial cell line (CCL-185™) was obtained from American Type Culture Collection (ATCC). The cell lines were cultured in DMEM supplemented with 10% FBS, 2 mM L-glutamine, 50 U/mL of penicillin, and 50 μg/mL of streptomycin. The cells were maintained in a 5% CO_2_ humidified incubator at 37 °C.

THP-1 cells (TIB-202^TM^) were purchased from the ATCC and using an RPMI 1640 medium supplemented with 10% FBS, 2 mM L-glutamine, 50 U/mL penicillin, and 50 μg/mL streptomycin at 37 °C in a humidified 5% CO_2_ atmosphere. THP-1 macrophage differentiation was induced via a 24 h exposure to 10 ng/mL of PMA in DMSO. Cells used for the resting condition were kept in the presence of PMA for 24 h in normal growth medium. The media was then changed to PMA-free RPMI during a resting stage for another 24 h. PMA-treated macrophages were maintained in a 5% CO_2_ humidified incubator at 37 °C and ready to use for the experiments.

### 2.8. Cell Viability Assay

Cytotoxicity of the BR extract and its active compounds against A549 cells and PMA-treated THP-1 macrophages was determined using 3-(4,5-dimethylthiazol-2-yl)-2,5-diphenyltetrazolium bromide (MTT) assay. Briefly, the A549 (3 × 10^3^ cell/well) and THP-1 (6.5 × 10^3^ cells/well) were treated with increasing concentrations of BR extract (0–200 μg/mL) or C3G and P3G (0–20 μg/mL) in culture medium or culture medium alone (vehicle control) for 24 and 48 h. Following the extract or active compound treatment, the cells were incubated with 10 μL of 0.5 mg/mL MTT in PBS for 4 h. The culture supernatant was then removed, and the culture was resuspended with 200 μL of DMSO to dissolve the MTT formazan crystals. The absorbance was measured at 540 and 630 nm using a UV−visible spectrophotometer. The assay was performed in triplicate at each concentration. Cells’ viability was calculated compared to control and interpreted as the percentage of control.

### 2.9. Determination of Cytokine Secretion by Enzyme-Linked Immunosorbent Assay (ELISA)

Firstly, A549 cells (3 × 10^5^ cell/well) or PMA-treated THP-1 macrophages (6.5 × 10^5^ cell/well) were seeded in a 6-well-plate overnight. After that, the cells were pre-treated with various concentrations of BR extract or active compounds for 4 h and then exposed to SARS-CoV-2 Spike Glycoprotein S1 at the concentration of 100 ng/mL. The cultured supernatants were collected and kept frozen at −80 °C for further use in determining of cytokine levels by ELISA testing. Secretions of IL-6, IL-1β, and IL-18 cytokines in the cultured supernatant were determined using an ELISA kit (Biolegend, San Diego, CA, USA) as per the manufacturer’s instruction and the absorbance was measured at 450 and 570 nm. The cytokine secretions in the cultured medium were calculated and compared for each standard curve.

### 2.10. Expression of NLRP3, IL-6, IL-1β, and IL-18 Genes by Reverse Transcription-Polymerase Chain Reaction (RT-qPCR) Analysis

Interleukin-6 (*IL-6)* primer sequence was supplied from Bio Basic Canada Inc., Markham, ON, Canada. Nucleotide-binding oligomerization domain-like receptor containing pyrin domain 3 (*NLRP3*), interleukin-1β (*IL-1β*), interleukin-18 (*IL-18*), and glyceraldehyde 3-phosphate dehydrogenase (*GAPDH*) primer sequences were supplied from Humanizing Genomics Macrogen, Geumcheon-gu, Seoul, Korea [31,32,33,34]. All primer sequences used in this study are shown in Table 1.

In order to determine inflammatory gene expressions (*NLRP3*, *IL-6*, *IL-1β* and *IL-18*), A549 and THP-1 were pre-treated with various concentrations of BR extract, C3G, or P3G for 4 h and then exposed to 100 ng/mL of SP. After 3 h of incubation, total mRNA was isolated using TRI reagent^®^. The concentration and purity of total RNA were detected using NanoDrop™ 2000/2000c Spectrophotometers (Thermo Fisher Scientific, Waltham, MA, USA) as the A260/A280 values > 1.8 indicated the appropriate purity of RNA. The cDNA was obtained via reverse transcription using a Mastercycler^®^ nexus gradient machine (Eppendorf, Hamburg, Germany). Quantitative real-time PCR technique was applied using a qRT-PCR ABITM 7500 Fast & 7500 Real-Time PCR machine (Thermo Fisher Scientific, Waltham, MA, USA). Gene expressions were analyzed using QuantStudio 6 Flex real-time PCR system software (Applied Biosystems, Waltham, MA, USA). The 2^−ΔΔCT^ method with normalization to GAPDH and controls was used for calculation of results.

### 2.11. Nuclear Extraction and NF-kB Activity Assay

In order to prepare the nuclear extract for the determination of NF-kB activity in SP-induction cells, the nuclear extract method was followed according to the previously described protocol [35]. Briefly, A549 and PMA-treated THP-1 cells were seeded at 2 × 10^6^ cells per culture dish overnight and then were pre-treated with various concentrations of BR extract, C3G, or P3G for 4 h and then exposed to 100 ng/mL of SP. After 3 h of incubation, the treated cells were collected and washed twice with ice-cold PBS. The cell pellet was suspended with 400 μL of lysis buffer. The cells were allowed to swell on ice for 20 min, after which 15 μL of 10% of Nonidet P-40 was added. The tubes were agitated on a vortex for 15 s and centrifuged at 12,000 rpm for 30 s. The supernatant was collected and was representative of the cytoplasm extract. The nuclear pellets were suspended in ice-cold nuclear extraction buffer with intermittent vortex for 30 min. The nuclear extract was centrifuged at 14,000 rpm for 12 min, and the supernatant was collected and used to determine the nuclear proteins.

The NF-kB activity in the nuclear extracts was determined by DNA-binding activity of the transcription factor using NF-kB p65 Transcription Factor Assay Kit (ab133112, Abcam, Cambridge, UK). Briefly, the nuclear extraction proteins at 15 μg/mL were added to the well-coated dsDNA templates carrying NF-κB response elements and incubated at 4 °C overnight. Positive control (provided with kit), and nonspecific binding samples were also incubated on the plate. After washing, the wells were treated with primary antibody (anti-NF-κB) for 1 h and secondary antibody (goat-anti-rabbit HRP) for 1 h. Then, the developing and stopping reagent was added, absorbance was read at 450 nm, and the readings for nonspecific binding were subtracted from each treatment. The proportional change in activity of each test sample relative to the average of the untreated samples was determined.

### 2.12. Western Blot Analysis

In order to determine the effects of BR extract and active compounds on inflammasome signaling proteins in SP-induced inflammation, A549 cells and PMA-treated THP-1 macrophages were pre-treated with various concentrations of BR extract and active compounds for 4 h and then exposed to 100 ng/mL of SP. After 3 h of incubation, cells were collected and lysed using RIPA buffer. The protein concentration was determined using the Bradford method. The whole-cell lysate was subjected to 12% SDS-PAGE. Separated proteins were transferred into nitrocellulose membranes. Membranes were blocked with 5% non-fat dried milk protein in 0.5% TBS-tween. After that, the membranes were washed twice with 0.5% TBS-Tween. Then, membranes were further incubated overnight with the primary antibody at 4 °C. Next, the membranes were washed 5 times with 0.5% TBS-Tween followed by incubating with horseradish peroxidase-conjugated anti-mouse or rabbit-IgG depending on the primary antibody at room temperature for 2 h and were then washed 5 times with 0.5% TBS-Tween. Bound antibodies were detected using the chemiluminescent detection system and then exposed to X-ray film (GE Healthcare Ltd., Little Chalfont, UK). Equal values of protein loading were confirmed as each membrane was stripped and re-probed with anti-b-actin antibody. Band density levels were analyzed using IMAGE J 1.410.

### 2.13. Statistical Analysis

All data are presented as mean ± standard deviation (S.D.) values. Statistical analysis was performed with SPSS 21.0 (IBM, New York, NY, USA) using independent t-test and one-way ANOVA with Tukey’s test. Statistical significance was determined at *p* < 0.05, and *p* < 0.01.

## 3. Results

### 3.1. Anthocyanin-Rich Fractions Prepared from Black Rice Germ and Bran (BR Extract) Contain Peonidin-3-O-glucoside (P3G) and Cyanidin-3-O-glucoside (C3G) as Major Anthocyanin Compounds

**Figure 1:**
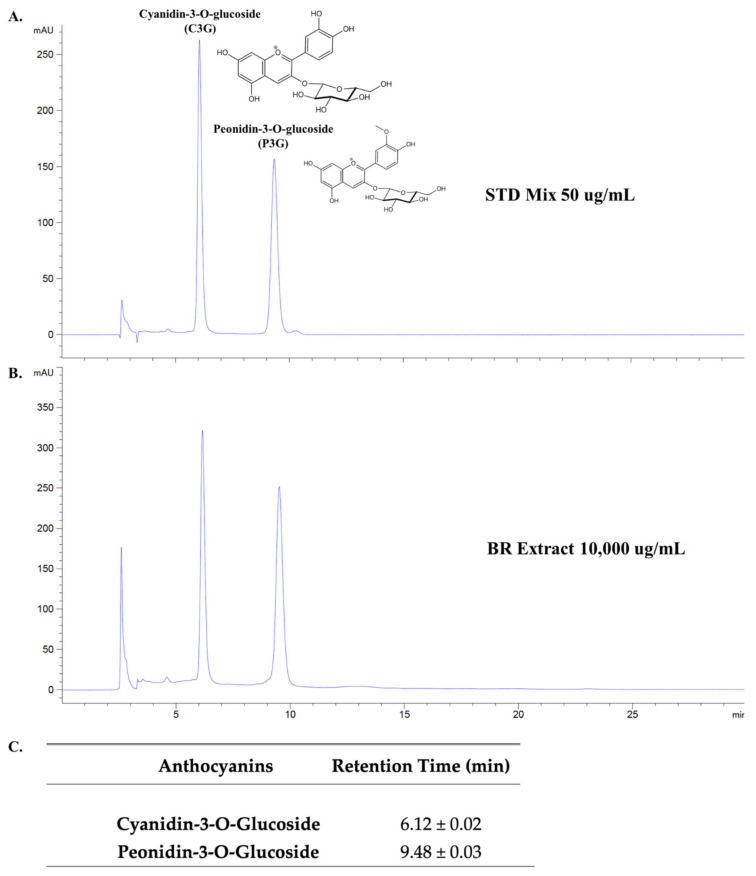
HPLC profile of anthocyanin-rich fraction of black rice germ and bran. HPLC chromatogram of active anthocyanin standards (C3G and P3G) according to their retention time at a concentration of 50 μg/mL for each standard (**A**). HPLC chromatogram of C3G and P3G-rich fraction from black rice germ and bran (BR extract) at 10,000 μg/mL (**B**). The mean and standard deviation (SD) of retention time (*n* = 3) for C3G and P3G compounds based on HPLC chromatogram (**C**). The first peaks of chromatogram in (**A**,**B**) are the solvent peaks (retention time = 2.61 ± 0.01 min). The HPLC chromatogram were evaluated using reversed-phase C18 column. The mobile phase was composed of mobile phase A (0.4% trifluoroacetic acid in water) and mobile phase B (0.45% trifluoroacetic acid in acetonitrile) under isocratic condition. The detection wavelength was 520 nm. The flow rate was set to 1.0 mL/min. HPLC, High-performance liquid chromatography; STD, Standard; BR extract, C3G and P3G-rich fraction of black rice germ and bran.

In this study, the percentage yield of final BR extract powder was 3.31 ± 0.98% (*w*/*w*). At first, the separate chromatograms of C3G and P3G have been done to identify the retention time of the respective anthocyanin standards as is shown in Figure S1. The main active compounds presented in the BR extract were identified and quantified using the HPLC technique. Two anthocyanin compounds that had previously been reported as the major anthocyanins of pigmented rice were C3G and P3G [19,20], and are shown in the HPLC chromatogram as the standards used in this study (Figure 1A). As expected, these two anthocyanins were identified in our BR extract. The phytochemical characteristics of the BR extract are shown in Table 2. According to the results, the total anthocyanin content of the BR extract accounted for 105.24 ± 6.66 mg/g extract. When the amount of the anthocyanins in the BR extract was calculated based on the HPLC data, it was found that the BR extract contained higher amounts of P3G (52.70 ± 1.80 mg/g extract) than C3G (33.04 ± 1.65 mg/g extract). In accordance with this result, it can be inferred that C3G and P3G were the major anthocyanins found in the BR extract.

### 3.2. BR Extract and Its Active Compounds, P3G and C3G, Displayed No Cytotoxicity in A549 Lung Cells and THP-1 Macrophages

**Figure 2:**
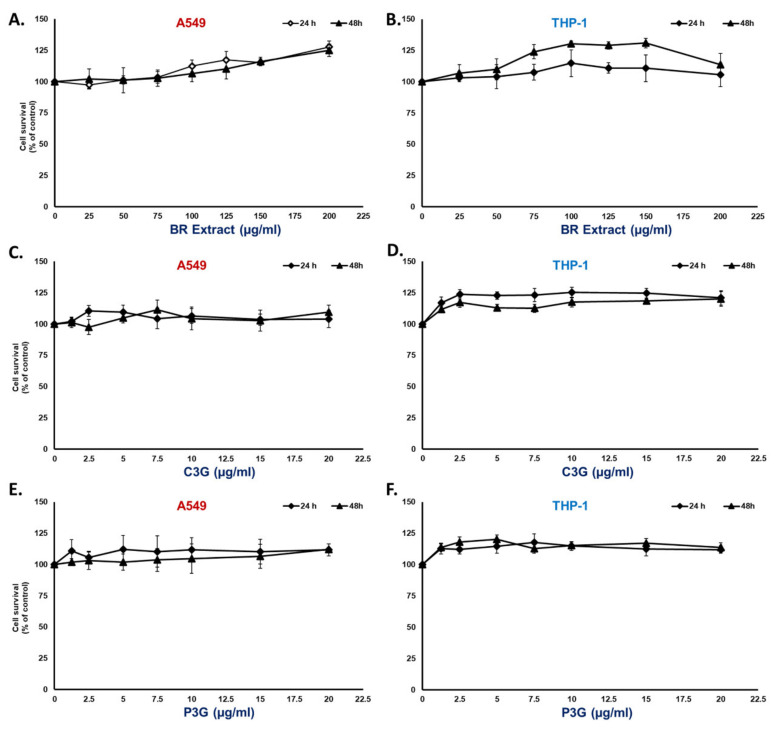
Cytotoxicity profile of BR extract and its active compounds on A549 lung cells and differentiated THP-1 macrophages. A549 (at 3 × 10^3^ cell/well) and PMA-treated THP-1 macrophage (6.5 × 10^3^ cell/well) were treated with BR extract at the concentration of 0–200 μg/mL (**A**,**B**), or the active compound; C3G (**C**,**D**); and P3G (**E**,**F**) at the concentration of 0–20 μg/mL for 24 h and 48 h. Cell viability was determined using MTT assay. Data are presented as mean ± S.D. values of at least three independent experiments. PMA, phorbol 12-myristate 13-acetate; MTT, 3-(4,5-dimethylthiazol-2-yl)-2,5-diphenyltetrazolium bromide.

Prior to the determination of the anti-inflammatory properties of the BR extract and its active compounds, we first examined whether the concentration was toxic to the cell lines that we used in this study. By employing the MTT assay shown in Figure 2 for A549 cells, we observed no cytotoxicity of the BR extract at concentrations of 0–200 μg/mL (Figure 2A) and 0–20 μg/mL for the active compounds C3G (Figure 2C) and P3G (Figure 2E) when the cells were treated for 24 h and 48 h. For the THP-1 macrophage, the concentration of phorbol 12-myristate 13-acetate (PMA) used for THP-1 macrophage differentiation was at a dosage of 10 ng/mL, which was the optimal concentration that did not cause cytotoxicity and was sufficient to differentiate the THP-1 cells (data not shown). According to the MMT results, both the BR extract and the active compounds (Figure 2B), C3G (Figure 2D) and P3G (Figure 2F), exhibited no cytotoxic effect at concentrations of 0–200 μg/mL for the BR extract and 0–20 μg/mL for the active compounds when the A549 cells and THP-1 macrophages were treated for 24 h and 48 h.

### 3.3. Spike Glycoprotein S1 (SP) Induced an Inflammatory Response by Upregulating the Inflammatory Gene Expressions and Increasing Cytokine Secretions in A549 Cells and THP-1 Macrophages

To validate the inflammatory response conditions in both A549 lung cells and PMA-treated THP-1 macrophages, we first determined the optimal time required for an inflammatory response upon SP induction. A concentration of SP at 100 ng/mL was chosen from the review literature of previous studies that were investigated on inflammatory responses in lung epithelial cells [7,36], monocytes, or macrophage cells [3,5]. This concentration was then confirmed in our own laboratory. The optimal time response was established by determining an increase in mRNA (*IL-6*, *NLRP3*, *IL-1β*, and *IL-18*) and protein levels (IL-6, Il-1β, and IL-18). These genes were chosen based on the intermediate gene and final cytokine products that were recognized during inflammatory responses, as IL-6 and NLRP3 and their related cytokines (Il-1β and IL-18) are the major immune components in immune response stimulation upon pathogen infection and inflammatory responses [13,37].

**Figure 3:**
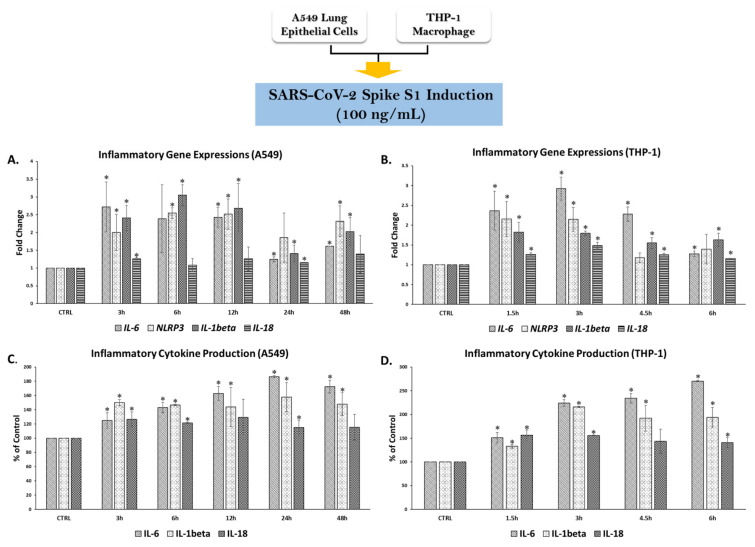
Establishing the inflammatory response upon spike S1 glycoprotein induction in A549 lung cells and THP-1 macrophages. PMA-treated THP-1 macrophage (6.5 × 10^5^ cell/well) and A549 (at 3 × 10^5^ cell/well) were seeded in 6-well plates and incubated overnight, then challenged with 100 ng/mL of spike glycoprotein S1 (SP) for 0–48 h for A549 and 0–6 h for THP-1 macrophage and the supernatants were collected and the cells were extracted for total RNA using TRIzol^®^ reagent. The fold-change of inflammatory gene expressions (*NLRP3*, *IL-1β*, and *IL-18*) in response to SP induction in A549 cells (**A**) and in THP-1 macrophages (**B**) were determined using RT-PCR. The inflammatory cytokine secretions (IL-6, IL1β, and IL-18) in A549 cells (**C**) and in THP-1 macrophages (**D**) upon SP induction were determined using ELISA. Data are presented as mean ± S.D. values of at least three independent experiments. * p < 0.05 vs. non-treated control group. SARS-CoV-2, severe acute respiratory syndrome coronavirus 2; IL-6, Interleukin-6; NLRP3, Nucleotide-binding oligomerization domain-like receptor containing pyrin domain 3; IL-1β, interleukin-1β; IL-18, interleukin-18.

In Figure 3, the time points for SP-induced inflammation in A549 lung cells were 0, 3, 6, 12, 24, and 48 h after SP induction. The inflammatory genes in A549 cells were significantly upregulated at 3 h after SP induction for all candidate inflammatory genes (Figure 3A, *p* < 0.05). The downregulation of inflammatory genes was observed at 24 h and 48 h of SP induction. Remarkably, the inflammatory gene responses coincided with the cytokine secretions in A549 cells as significant increases in all cytokine levels were observed at 3 h after SP induction (Figure 3C, *p* < 0.05). For PMA-treated macrophages, the time points for SP-induced inflammation were 0, 1.5, 3, 4.5, and 6 h after SP induction. The inflammatory gene expressions in THP-1 cells were significantly upregulated at 1.5 h after SP induction and reached the highest expression at 3 h after the induction for all candidate inflammatory genes (Figure 3B, *p* < 0.05). Downregulation of the inflammatory genes was observed at 6 h of SP induction. The inflammatory gene responses coincided with the cytokine secretions in A549 cells as significant increases in cytokine levels were observed at 1.5 h and reached the highest point at 3 h after SP induction for all cytokines (Figure 3D, *p* < 0.05). Therefore, we determined that the optimal time for SP induction to sufficiently initiate an inflammatory response was 3 h after the induction for both A549 lung cells and PMA-treated macrophages. Subsequently, that specific time of incubation was further used to determine the inhibitory effects of the BR extract and its active compounds upon SP-induced inflammation.

### 3.4. BR Extract and the Active Anthocyanins C3G and P3G Exhibited Anti-Inflammatory Properties upon SP Induction in A549 Cells and THP-1 Macrophages

#### 3.4.1. Effects of BR Extract on SP Induction in A549 Cells and THP-1 Macrophages

**Figure 4:**
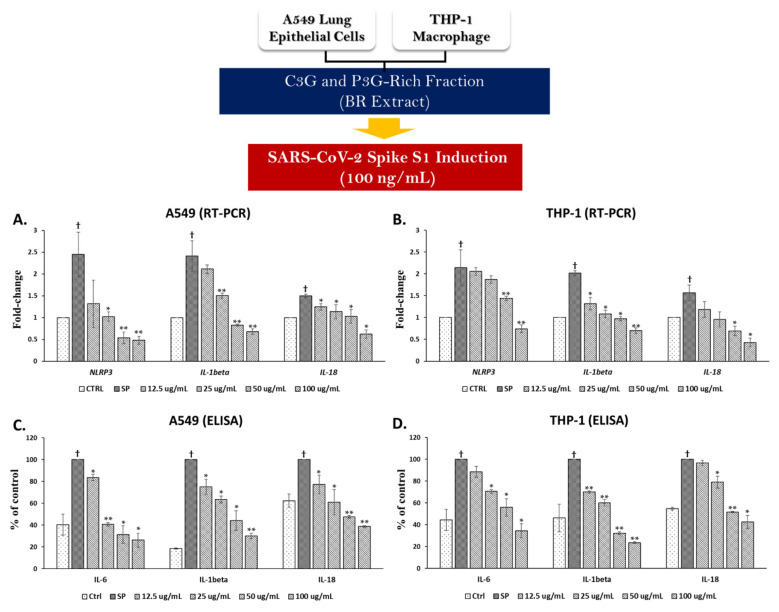
Anti-inflammation properties of BR extract upon SP induction in A549 lung cells and THP-1 macrophages. A549 (at 3 × 10^5^ cell/well) and PMA-treated THP-1 macrophage (6.5 × 10^5^ cell/well) were pre-treated with BR extract at the concentration of 0–100 μg/mL for 4 h, and then challenged with 100 ng/mL of SP for 3 h, then the supernatant was collected, and total RNA was extracted using TRIzol^®^ reagent. The fold-change inhibition of inflammatory gene expressions (*NLRP3*, *IL-1β*, and *IL-18*) in A549 cells (**A**) and in THP-1 macrophages (**B**) upon BR extract treatment were determined using RT-PCR. The effect of BR extract on the inhibition of cytokine production (IL-6, IL1β, and IL-18) in A549 cells (**C**) and THP-1 macrophage (**D**) were determined using ELISA. Data are presented as mean ± S.D. values of at least three independent experiments. * p < 0.05, ** *p* < 0.01 vs. SP-induced control group. ^†^ *p* < 0.05 vs. non-treated control group. RT-PCR, Reverse transcription-polymerase chain reaction; ELISA, enzyme-linked immunosorbent assay.

After establishing the conditions for an SP-induced inflammatory response, we investigated the anti-inflammatory properties of the BR extract and its anthocyanin active compounds. In A549 cells, SP induction resulted in significant increases in mRNA levels of approximately 2.5-fold for *NLRP3* and *IL-1β*, and 1.5-fold for *IL-18* when compared with the non-SP control group (Figure 4A, *p* < 0.05). When A549 cells were pre-treated with the BR extract at concentrations of 12.5–100 μg/mL, the mRNA levels of the inflammatory genes were significantly decreased starting from 12.5 μg/mL to 100 μg/mL of the BR extract for *IL-18* (*p* < 0.05) and from 25 μg/mL to 100 μg/mL for the other inflammatory genes (*NLRP3* and *IL-1β*, *p* < 0.05).

SP exposure in A549 also resulted in significant increases in cytokine production by approximately 60% for IL-6, 80% for IL-1β, and 40% for IL-18 when compared with the non-SP control group (Figure 4C, *p* < 0.05). When the cells were pre-treated with the BR extract at concentrations of 12.5–100 μg/mL, decreases in the cytokine levels were observed for all cytokines, including IL-6, IL-1β, and IL-18, in a dose-dependent manner (*p* < 0.05).

Likewise, SP induction in PMA-treated macrophages resulted in significant increases in mRNA levels at approximately 2-fold for *NLRP3* and *IL-1β* and 1.6-fold for *IL-18*, when compared with the non-SP control group (Figure 4B, *p* < 0.05). When the cells were pre-treated with the BR extract, mRNA levels of the inflammatory genes were significantly decreased starting from 12.5 μg/mL to 100 μg/mL of the BR extract for *IL-1β* (*p* < 0.05) and from 50 μg/mL to 100 μg/mL for the other inflammatory genes (*NLRP3* and *IL-18*, *p* < 0.05). With regard to cytokine production, SP exposure in PMA-treated macrophages resulted in significant increases in cytokine levels at approximately 60% for IL-6 and IL-1β, and 50% for IL-18 when compared with the non-SP control group (Figure 4D, *p* < 0.05). When the cells were pre-treated with the BR extract, significant decreases in cytokine levels in a dose-dependent manner were observed from 12.5 μg/mL to 100 μg/mL in IL-1β (*p* < 0.05), and from 25 μg/mL to 100 μg/mL for IL-6 and IL-18 (*p* < 0.05). Overall, the BR extract was a broad cytokine inhibitor at mRNA and protein levels in both A549 and THP-1 cell lines.

#### 3.4.2. Effects of C3G and P3G on SP Induction in A549 Cells and THP-1 Macrophages

**Figure 5:**
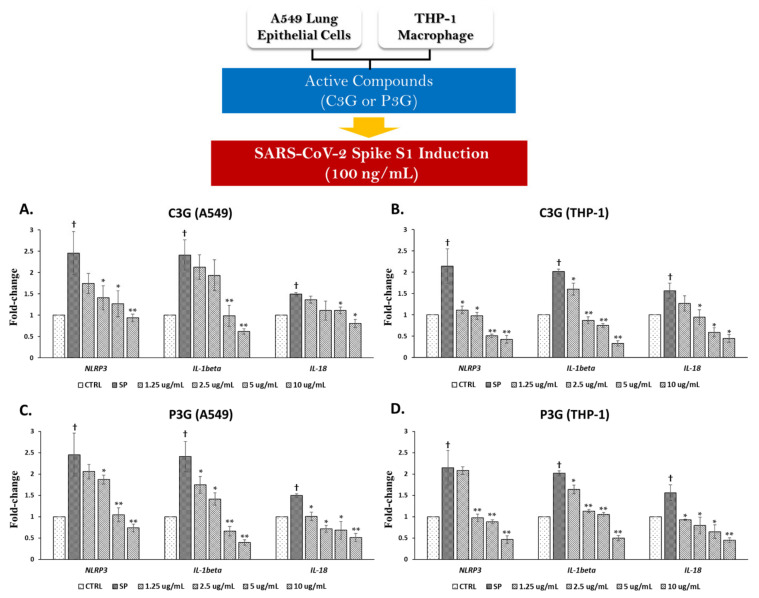
Cyanidin-3-O-glucoside (C3G) and peonidin-3-o-glucoside (P3G) suppressed the inflammatory gene expressions upon SP induction in A549 lung cells and THP-1 macrophages. A549 (at 3 × 10^5^ cell/well) and PMA-treated THP-1 macrophage (6.5 × 10^5^ cell/well) were pre-treated with the active compounds at the concentration of 0–10 μg/mL for 4 h, and then challenged with 100 ng/mL of SP for 3 h, then the total RNA was extracted using TRIzol^®^ reagent. The effect of C3G (**A**,**B**) and P3G (**C**,**D**) on fold-change inhibition of inflammatory gene expressions (*NLRP3*, *IL-1β*, and *IL-18*) in A549 cells and in THP-1 macrophages upon SP induction were determined using RT-PCR. Data are presented as mean ± S.D. values of at least three independent experiments. * p < 0.05, ** *p* < 0.01 vs. SP-induced control group. ^†^ *p* < 0.05 vs. non-treated control group.

**Figure 6:**
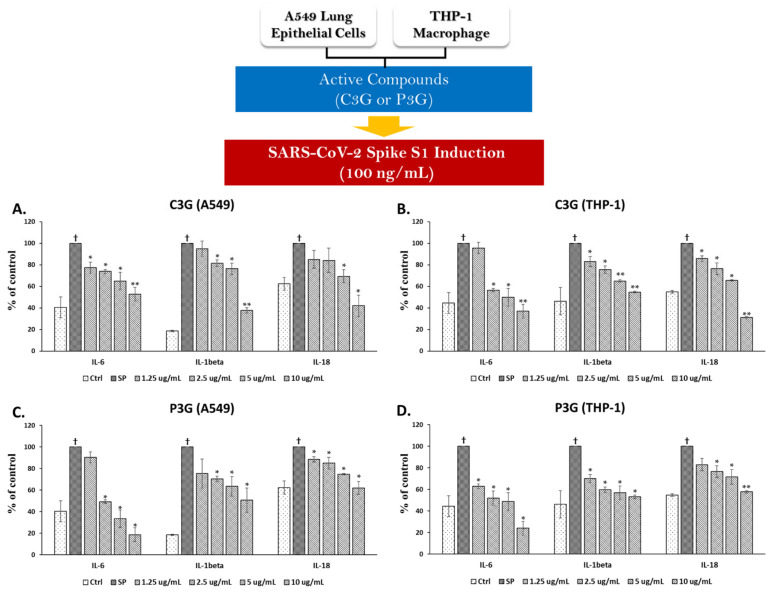
Cyanidin-3-O-glucoside (C3G) and peonidin-3-o-glucoside (P3G) ameliorated the SP-induced inflammation in A549 lung cells and THP-1 macrophages by inhibiting the cytokine secretions. A549 (at 3 × 10^5^ cell/well) and PMA-treated THP-1 macrophage (6.5 × 10^5^ cell/well) were pre-treated with the active compounds at the concentration of 0–10 μg/mL for 4 h, and then challenged with 100 ng/mL of SP for 3 h, then the supernatant was collected. The inhibitory effect of C3G (**A**,**B**) and P3G (**C**,**D**) on SP-induced inflammatory cytokine secretions (IL-6, IL1β, and IL-18) in A549 cells and in THP-1 macrophages were determined using ELISA. Data are presented as mean ± S.D. values of at least three independent experiments. * p < 0.05, ** *p* < 0.01 vs. SP-induced control group. ^†^ *p* < 0.05 vs. non-treated control group.

Since we found that the BR extract contained two major anthocyanins, we further examined the inhibitory effects of C3G and P3G on SP-induced inflammation. With regard to the inhibition of SP-induced inflammatory gene expression, C3G at concentrations of more than 1.25 μg/mL was able to significantly suppress the mRNA levels of inflammatory genes, including *NLRP3*, *IL-1β*, and *IL-18*, in both A549 cells and PMA-treated macrophages in a dose-dependent manner (Figure 5A,B, *p* < 0.05). Similarly, through exposure of SP on the A549 and THP-1 cells for 3 h, the data demonstrated that C3G could significantly inhibit the inflammatory cytokine levels (IL-6, IL-1β, and IL-18) in a dose-dependent manner (Figure 6A,B, *p* < 0.05).

Similar to C3G, P3G, which is another anthocyanin identified in the BR extract, exhibited inflammatory gene suppression for both A549 cells and THP-1 macrophages. The concentration of 1.25–10 μg/mL of P3G was found to be able to significantly suppress the mRNA levels of *NLRP3*, *IL-1β*, and *IL-18* in a dose dependent-manner (Figure 5C,D, *p* < 0.05). Additionally, P3G at the same range of concentration could significantly inhibit the inflammatory cytokine levels (IL-6, IL-1β, and IL-18) in a dose-dependent manner (Figure 6C,D, *p* < 0.05). Overall, it can be assumed that the inhibitory effects of the BR extract on SP-induced inflammation was due to the presence of two major anthocyanin compounds, C3G and P3G. As these two anthocyanins possess compatible anti-inflammation properties, they could both significantly inhibit the inflammatory response of both mRNA and protein levels when A549 and THP-1 macrophages were primed with 100 ng/mL of SP.

### 3.5. BR Extract and Its Active Anthocyanins, C3G and P3G, Exhibited Anti-Inflammatory Properties through Inhibition of NF-kB Transcriptional Activity and the NLRP3 Inflammasome Pathway in A549 and THP-1 Cell Lines

#### 3.5.1. Inhibitory Effects of BR Extract, C3G, and P3G on NF-kB Transcriptional Activity in A549 Cells and THP-1 Macrophages

**Figure 7:**
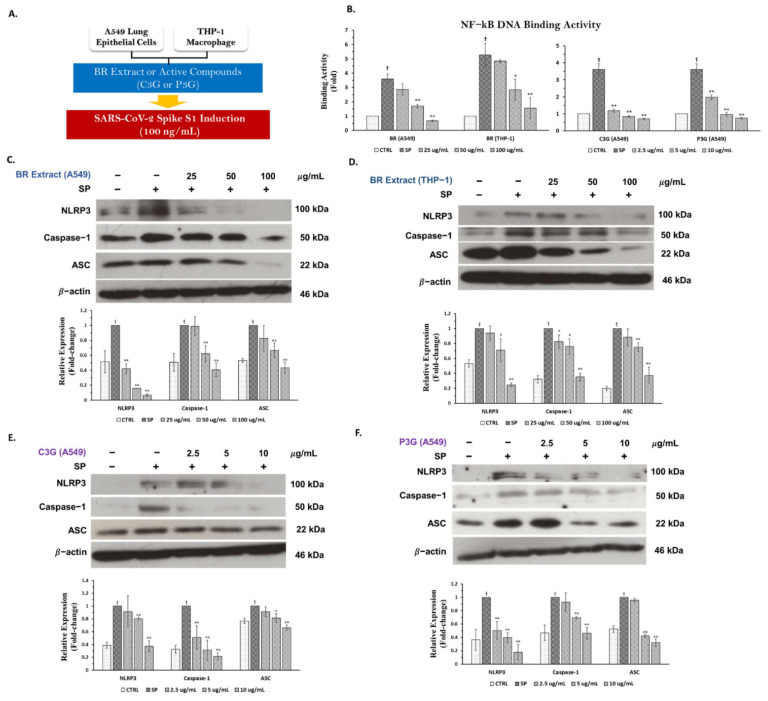
BR extract and its active compounds C3G and P3G exhibited anti−inflammatory properties through the inhibition of NLRP3 inflammasome pathway in A549 lung cells and THP−1 macrophages. A549 cells (at 3 × 10^5^ cell/well) and PMA−treated THP−1 macrophage (6.5 × 10^5^ cell/well) were pre−treated with the BR extract at the concentration of 0−100 μg/mL or C3G and P3G at the concentration of 0−10 μg/mL for 4 h, and then challenged with 100 ng/mL of SP for 3 h (**A**), then the cell lysates were collected. Cells were kept and determined for the inhibition of inflammasome machinery proteins including NLRP3, caspase−1, and ASC using Western blot analysis. The inhibitory effects of BR extract and active anthocyanins on NF−kB transcriptional activity in nuclear extracts (**B**). The inhibitory effects of the BR extract in A549 cells (**C**) and in THP−1 cells (**D**), C3G (**E**), and P3G (**F**) on NLRP3, caspase−1, and ASC proteins displayed in western blots and band density measurements. * p < 0.05, ** *p* < 0.01 vs. SP−induced control group. ^†^ *p* < 0.05 vs. non−treated control group.

Previously compiled data have indicated the significance of NF-kB activation as a priming signal for the canonical inflammasome pathway, and NF-kB nuclear translocation can promote the transcription of inflammasome-related genes including *NLRP3*, *IL-1β*, and *IL-18* [12,31,38]. Therefore, we examined whether the BR extract and its active anthocyanins could inhibit NF-kB activity upon SP-induced inflammation. According to the results shown in Figure 7B, the exposure of 100 ng/mL of SP for 3 h resulted in a significant increase in NF-kB activity as determined by the NF-kB transcriptional activity assay for both A549 and THP-1 cells. When we pre-treated the cells with the BR extract in A549 cells and THP-1 macrophages and its active anthocyanins (C3G and P3G) in A549 cells, a significant reduction in NF-kB activity was observed in a dose-dependent manner (*p* < 0.05).

#### 3.5.2. Inhibitory Effects of BR Extract, C3G, and P3G on NLRP3 Inflammasome Pathway in A549 Cells and THP-1 Macrophages

As NLRP3 inflammasome is the potential inflammatory pathway for inflammation-related COVID-19 infection [39,40], we sought to determine whether our BR extract and its anthocyanins could inhibit an inflammatory response through this pathway using the Western blotting technique. After SP (at 100 ng/mL) induction for 3 h in both A549 lung cells and PMA-treated THP-1 macrophages, the inflammasome machinery protein expressions, including NLRP3, capase-1, and ASC proteins, were significantly enhanced when compared with the non-SP control as is shown in the band density of Figure 7C,F (*p* < 0.05). When A549 cells and THP-1 macrophages were pre-treated with the BR extract at concentrations of 25–100 μg/mL, it was found that the BR extract could significantly attenuate SP-induced inflammation via downregulation of the protein’s expressions in inflammasome pathways (NLRP3, ASC, and capase-1) in both A549 cells and THP-1 macrophages, as is shown in Figure 7C,D (*p* < 0.05 for the scanned band densities). Similarly, when the cells were treated with C3G or P3G prior to SP-induction, both active anthocyanins could reduce the expression of inflammasome machinery protein for both A549 cells (Figure 7E,F, *p* < 0.05 for the scanned band densities) and THP-1 macrophages (data not shown). Overall, it can be concluded that the inhibitory effects of the BR extract and its active compounds on SP-induced inflammation were partly due to the inhibition of NF-kB activity and the NLRP3 inflammasome pathway through the suppression of those inflammasome machinery protein expressions (NLRP3, ASC, and caspase-1) in both A549 and THP-1 macrophages.

## 4. Discussion

Since December of 2019, the SARS-CoV-2 outbreak has been spreading with increasing degrees of severity and infectivity [6,41]. Currently, the primary concern for healthcare systems for SARS-CoV-2-infected patients is not only to prevent or limit the cytokine storm conditions, but also to deal with the inflammation-related post-acute COVID condition (long-COVID) that potentially results from the action of spike glycoprotein of SARS-CoV-2 and other inflammatory mediators on target tissues [1,9,14]. As of today, only few specific therapies are available and only indicated to use predominantly for anti-viral purposes [42,43]. Accordingly, the urgent solution is the use of dietary compounds that can be found abundantly in functional food, and these could be used conveniently in home therapies and to target the inflammatory responses that occur during the onset of cytokine storms and inflammation-related long-COVID syndromes [13,44].

Flavonoids are plant water-soluble secondary metabolites that are the most abundant polyphenols present in the human diet. Epidemiological evidence suggests that a high intake of flavonoid-containing plant foods is associated with lower risks of many non-communicable diseases such as cardiovascular disease, diabetes, and chronic inflammatory diseases [45]. Numerous dietary flavonoids, such as epigallocatechin gallate (EGCG), kaempferol, quercetin, curcumin, and anthocyanins, have been identified by molecular docking studies as effective anti-SARS-CoV-2 agents [25,39,46,47]. Flavonoids can be subdivided into four subclasses, anthocyanins, flavonols, flavones, and flavanones. Among the subclasses, anthocyanin compounds display diverse beneficial effects that include anti-inflammation activities [45,48]. Previously, black rice (*Oryza sativa* L.) and black rice extracts have been considered beneficial functional foods because of their high content in anthocyanins, especially in their outer layers as these phytochemicals mainly accumulate in the germ and bran [17,19]. In our study, we established an anthocyanin-rich fraction from black rice germ and bran (BR extract) using our previous extraction method [23]. We then determined the anthocyanin content of this fraction.

The total amount of anthocyanin in the BR extract was within the range of the reference amount of anthocyanin content that is usually reported in black rice germ and bran extracts (ranging from 28.4–142.62 mg/g extract) [22,49]. We also found that two major anthocyanins, which are peonidin-3-O-glucoside (P3G) and cyanidin-3-O-gluside (C3G), were abundantly found in the BR extract. Importantly, these two anthocyanidins were previously reported for their anti-oxidative stress and anti-inflammatory properties [50,51,52]. In our study, the BR extract, therefore, can be considered a potential candidate in the development of anti-inflammatory agents for treating SARS-CoV-2 spike protein-induced inflammatory responses.

SARS-CoV-2 is a positive-sense single-stranded RNA virus that primarily causes infection in the respiratory tract. It infects human cells through the spike glycoprotein, which binds to the angiotensin converting enzyme 2 (ACE2) receptor and is expressed on lung epithelial cells, endothelial cells, and innate immune cells such as monocytes and macrophages [7,8]. The mature spike glycoprotein is a heavily glycosylated trimer with each protomer composed of 1260 amino acids (residues 14-1273). The S1 subunit is composed of 672 amino acids (residues 14-685) and is organized into four domains including the receptor-binding domain (RBD) [3,5]. Many studies have shown that in addition to facilitating its fusion to the cell membrane, the location of the spike glycoprotein on SARS-CoV-2 also makes it a direct target for the immune systems of the host [1,9]. Specifically, the spike glycoprotein S1 subunit, which is the RBD and the subunit for ACE2 binding, was found to trigger an inflammatory response when stimulated in human peripheral blood mononuclear cells (PBMCs). Accordingly, a concentration of 100 ng/mL of S1 of the spike glycoprotein resulted in significant increases in cytokine production (TNFα, IL-6, IL-1β and IL-8) [3,5,53]. Moreover, it was hypothesized that the spike protein could imitate the action of pathogen-associated molecular pattern (PAMP) and stimulate THP-1 macrophages and lung epithelial cells (A549), leading to activation of an inflammatory response via the induction of inflammatory cytokines and chemokines [54,55]. These results coincided with those of our study, as 100 ng/mL of SP was able to trigger an inflammatory response at both gene (*NLRP3, IL-1β*, and *IL-18*) and protein levels (IL-6, IL-1β, and IL-18) in the activated THP-1 macrophages as well as in A549 lung epithelial cells.

It has been hypothesized that during the pulmonary hyperinflammatory response, alveolar macrophages and lung epithelial cells can express and activate NLRP3 inflammasome machinery proteins, which have been identified as one of the most detrimental signaling molecules in many inflammatory-related lung diseases. For this reason, it is possible that NLRP3 and its downstream inhibition can be considered a novel target in the development of supportive therapies [39,40,41]. Many studies have shown that the inhibition of the NLRP3 inflammasome inflammatory pathway reduced the release of pro-inflammatory cytokines in SARS-CoV-2 infected immune cells and lung epithelial cells. The use of the small molecule MCC950, a specific inhibitor of NLRP3 inflammasome activity, alleviated COVID-19 like pathology in hACE2 transgenic mice [13,56,57,58]. In clinical settings, the pro-inflammatory cytokine IL-1β was elevated in plasma taken from hospitalized SARS-CoV-2 infected patients, while its associated signaling pathway seemed to drive SARS-CoV-2 pathogenicity [1,14,59].

In turn, black rice, as well as its active compounds, anthocyanins, have been studied for their anti-inflammatory properties, especially with regard to the inhibition of the NLRP3 inflammasome pathway. Anthocyanins from *Hibiscus syriacus* L. inhibited LPS/ATP-induced NLRP3 inflammasome by inhibiting the NF-κB signaling pathway and leading to the inhibition of IL-1β and IL-18 release [60]. Another study found that, C3G from black rice possessed anti-inflammatory properties in lipopolysaccharide-induced RAW 264.7 cells and carrageenan-induced inflammation in air pouches in BALB/c mice through the suppression of the proinflammatory cytokines, TNF-α and IL-1β, mechanistically via regulating NF-κB and MAPK activation [50]. Moreover, a daily dosage of 320 mg of anthocyanin capsules administration resulted in a decrease in mRNA expression of NLRP3 inflammasome components (*caspase-1*, *IL-1β*, and *IL-18*) in PBMCs and the plasma levels of IL-1β and IL-18 in NAFLD patients when compared with the controls [61]. Therefore, in this study, we investigated the inhibitory effects of the BR extract and its active compounds on SP-induced inflammation by determining the inhibition of the final products of the NLRP3 inflammasome pathway with regard to both gene and protein levels, and to ultimately determine the inhibition of the inflammasome signaling pathway. According to our results, the BR extract exhibited anti-inflammatory properties upon SP-induction in A549 lung epithelial cells and in PMA-treated THP-1 macrophages through the inhibition of *NLRP3, IL-1β*, and *IL-18* genes, as well as via their protein expressions. These inhibitory effects were partly due to the presence of two major anthocyanins in the extract, C3G and P3G, as has been confirmed by the treatment of these compounds in both cell lines under SP-induced conditions.

Additionally, the data from Figure 4 and Figure 6 could suggest the synergistic effect of two anthocyanins. With regard to the concentration of two major anthocyanins in BR extract, 100 microgram of BR extract would contain approximately 3.3 micrograms of C3G and 5.27 micrograms of P3G. When comparing the inhibition of cytokine secretions (IL-6, IL-1β, and IL-18) of BR extract at 100 μg/mL with the inhibition by two active anthocyanins (C3G and P3G), it was found that the percentage inhibition of BR extract containing both C3G and P3G exhibited more potent anti-inflammatory properties than either C3G or P3G alone for both A549 and THP-1 cells.

IL-1β and IL-18 cytokine secretions are primarily initiated by inflammasomes that represent multiprotein signaling platforms responsible for the coordination of early antimicrobial host defense [31,37]. The NLRP3 inflammasome is comprised of the NLRP3 sensor, caspase-1, and the adaptor molecule apoptosis-associated speck-like protein containing a caspase recruitment domain (ASC). When activated, matured caspase-1 cleaves pro-IL-1β and pro-IL-18 into mature IL-1β and IL-18, respectively, which are then secreted [62,63]. In this study, we further investigated whether the BR extract and its active compounds could suppress the inflammatory responses via inhibition of the inflammasome pathway. It was found that the BR extract and its active anthocyanin compounds attenuated the SP-induced inflammatory response in both lung epithelial cells and activated macrophages through the inhibition of inflammatory genes and cytokine secretions mechanistically via the downregulation of the NLRP3 inflammasome pathway machinery proteins including the expression levels of NLRP3, ASC, and caspase-1.

The cytokine storm associated with ARDS, which is initiated by SARS-CoV-2 infection, can lead to toll-like receptor (TLR) signaling and NF-κB activation [54,64]. Previous studies have reported that the spike protein appeared to share antigenic epitopes with human molecular chaperons resulting in autoimmunity and can activate TLRs leading to a release of inflammatory cytokines [65]. The spike protein of SARS-CoV-2 is a potent viral PAMP and is sensed by TLR of the host, which stimulates macrophages, monocytes, and lung epithelial cells leading to the activation of the NF-κB pathway and induction of inflammatory cytokines and chemokines [7,10]. Thus, in this study we also examined the inhibitory effects of the BR extract and its active compounds on NF-κB activation through the inhibition of NF-κB transcriptional activity upon SP-induction in both cell lines. From the results of this study, it can be assumed that SP possibly interacted with TLR and triggered inflammatory responses through the NF-κB/NLRP3 inflammasome axis resulting in increased expressions of IL-1β and IL-18, while the BR extract, as well as its active anthocyanins, could suppress inflammatory responses upon spike glycoprotein S1 induction through this exact mechanism.

This study employed black rice germ and bran, which is rich in anthocyanins (C3G and P3G), to attenuate the inflammatory responses induced by the spike glycoprotein S1 subunit of SARS-CoV-2 via the inhibition of the NLRP3 inflammasome pathway. According to the findings, consumption of functional food such as black rice germ and bran can be encouraged for home therapy as a preventive measure for inflammation-related after-effects of SARS-CoV-2 infection, especially in the vulnerable age group (more than 65 years) that are susceptible to many chronic inflammatory diseases. This study also emphasized the important NLRP3 inflammasome pathway as a potential target for SARS-CoV-2 induced inflammation. However, further studies on the inhibition of upstream molecular proteins such as TLRs- and MyD88-dependent pathways should also be done to fully elucidate the anti-inflammatory mechanism of C3G and P3G-rich fraction of black rice germ and bran. Additionally, the anti-inflammatory properties of black rice germ and bran in a clinical setting needs to be further investigated.

## 5. Conclusions

Overall, our study identified two major anthocyanins, C3G and P3G, in black rice germ and bran and demonstrated the anti-inflammatory properties of black rice germ and bran and its active anthocyanins upon the spike protein S1 subunit of SARS-CoV-2 induction. Regarding the anti-inflammatory mechanism, the C3G and P3G-rich fraction of black rice germ and bran counteracted NF-κB activity and the NLRP3 inflammasome pathway, leading to the inhibition of inflammatory genes and cytokine secretions in lung epithelial cells and activated macrophages. Black rice germ and bran can be recognized as anthocyanin-rich dietary compounds that possesses anti-inflammation properties and can be further used as nutraceutical products for human health.

The outcome of this study elucidates the importance of preventing inflammation-related SARS-CoV-2 infection by targeting the NLRP3 inflammasome pathway, along with the applicability of anthocyanins from black pigmented rice as potential candidates in the development of supportive therapies derived from dietary compounds. Black rice germ and bran and their bioactive products could potentially be employed in home therapies by targeting the involved cytokines during SARS-CoV-2-induced inflammation, as well as by also targeting the after-effects of the inflammation-related long-COVID phenomenon.

## Figures and Tables

**Table 1:** Primer sequences used in this study for determination of gene expressions by RT-qPCR analysis [31,32,33,34].

Gene Product	Primer Sequence
*NLRP3*	Forward: 5′ AAG GGC CAT GGA CTA TTT CC 3′ Reverse: 5′ GAC TCC ACC CGA TGA CAG TT 3′
*IL-6*	Forward: 5′-ATG AAC TCC TTC ACA AGC-3′ Reverse: 5′-GTT TTC TGC CAG TGC CTC TTT G-3′
*IL-1β*	Forward, 5′ ATG ATG GCT TAT TAC AGT GGC AA 3′ Reverse, 5′ GTC GGA GAT TCG TAG CTG GA 3′
*IL-18*	Forward, 5′ AAA CTA TTT GTC GCA GGA ATA AAG AT 3′ Reverse, 5′ GCT TGC CAA AGT AAT CTG ATT CC 3′
*GADPH*	Forward, 5′ TCA ACA GCG ACA CCC AC 3′ Reverse, 5′ GGG TCT CTC TCT TCC TCT TGT G 3′

**Table 2:** Phytochemical characteristics of anthocyanin-rich fractions derived from black rice germ and bran (BR extract).

Phytochemicals	mg/g Extract
Total phenolic contents	115.25 ± 10.55
Total flavonoids	68.23 ± 6.84
Total anthocyanins	105.24 ± 6.66
Cyanidin-3-glucoside (C3G)	33.04 ± 1.65
Peonidin-3-glucoside (P3G)	52.70 ± 1.80

Data are presented as mean ± S.D. values of at least three independent experiments.

## Data Availability

Not applicable.

## Supplementary Materials

The following supporting information can be downloaded at: https://www.mdpi.com/article/10.3390/nu14132738/s1, Figure S1: Separate HPLC chromatograms of C3G, P3G, and standard mix.
