# Supplementary material for: Cyanidin-3-O-glucoside and Peonidin-3-O-glucoside-Rich Fraction of Black Rice Germ and Bran Suppresses Inflammatory Responses from SARS-CoV-2 Spike Glycoprotein S1-Induction In Vitro in A549 Lung Cells and THP-1 Macrophages via Inhibition of the NLRP3 Inflammasome Pathway
Source: Nutrients. 2022 Jun 30;14(13):2738. doi: 10.3390/nu14132738 (PMC9268823; doi:10.3390/nu14132738)
Supplement: Supplementary file 1 [file nutrients-14-02738-s001.zip › nutrients-1791859-supplementary.pdf]

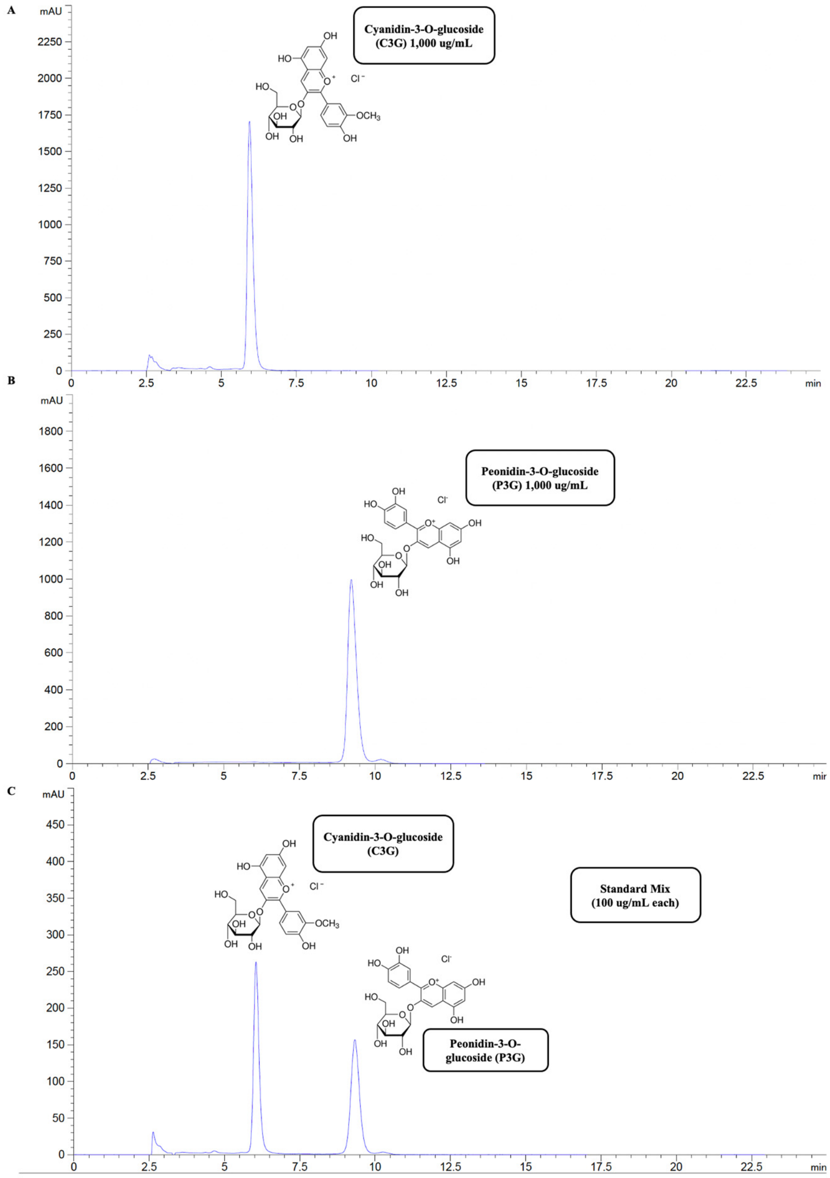

Figure S1. HPLC chromatograms of C3G, P3G, and standard mix. HPLC chromatogram of standard of C3G at 1,000 ug/mL (A) and P3G at 1,000 ug/mL (B) and Standard mix of C3G and P3G anthocyanins at 100 ug/mL for each standard (C) as determined by HPLC technique. The HPLC chromatogram were evaluated using reversed-phase C18 column. The mobile phase was composed of mo-bile phase A (0.4% Trifluoroacetic acid in water) and mobile phase B (0.45% Trifluoroacetic acid in acetonitrile) under isocratic condition. The detection wavelength was 520 nm. The flow rate was set to 1.0 mL/min.
